# Supplementary material for: Structural basis of malaria parasite phenylalanine tRNA-synthetase inhibition by bicyclic azetidines
Source: Nat Commun. 2021 Jan 12;12:343. doi: 10.1038/s41467-020-20478-5 (PMC7803973; doi:10.1038/s41467-020-20478-5)
Supplement: Supplementary file 1 — Supplementary Information [file 41467_2020_20478_MOESM1_ESM.pdf]

Structural basis of malaria parasite phenylalanine tRNA-synthetase inhibition by bicyclic azetidines

Manmohan Sharma[1#,2], Nipun Malhotra[1#], Manickam Yogavel[1#], Karl Harlos[3], Bruno Melillo[4,5], Eamon Comer[4], Arthur Gonse[4], Suhel Parvez[2], Branko Mitasev[6], Francis G. Fang[6], Stuart L. Schreiber[4,7] and Amit Sharma[1,8*]

1. Molecular Medicine, Structural Parasitology Group, International Centre for Genetic Engineering and Biotechnology, Aruna Asaf Ali Marg, New Delhi 110067, India.

2. Department of Medical Elementology and Toxicology, Jamia Hamdard University, New Delhi 110062, India.

3. Division of Structural Biology, Welcome Centre for Human Genetics, University of Oxford, Oxford OX3 7BN, England.

4. Chemical Biology and Therapeutics Science Program, Broad Institute of Harvard and MIT, 415 Main Street, Cambridge, Massachusetts 02142, USA.

5. Department of Chemistry, The Scripps Research Institute, 10550 North Torrey Pines Road, La Jolla, California 92037, USA.

6. Eisai Inc., 35 Cambridgepark Drive Suite 200, Cambridge, Massachusetts 02140, USA.

7. Department of Chemistry and Chemical Biology, Harvard University, 12 Oxford Street, Cambridge, Massachusetts 02138, USA.

8. National Institute of Malarial Research, Sector 8 Dwarka, New Delhi 110077, India.

[#]equal authorship

*Corresponding author, directornimr@gmail.com

Supplementary Methods

General considerations

Oxygen and/or moisture sensitive reactions were carried out in oven or flame-dried glassware under nitrogen atmosphere. All reagents and solvents were purchased and used as received from commercial vendors or synthesized according to cited procedures. Yields refer to chromatographically and spectroscopically pure compounds, unless otherwise stated. Flash chromatography was performed using 20-40 μm silica gel (60 Å mesh) on a Teledyne Isco Combiflash Rf. Analytical thin layer chromatography (TLC) was performed on 0.2 mm or 0.25 mm silica gel 60-F plates and visualized by UV light (254 nm). NMR spectra were recorded on Bruker 300 (^{1}H, 300 MHz; ^{13}C, 75 MHz) or 400 (^{1}H, 400 MHz; ^{13}C, 100 MHz) or Varian 400MR (^{1}H, 400 MHz; ^{13}C, 100 MHz) spectrometers at 300 K unless otherwise noted. Chemical shifts are reported in parts per million (ppm) relative to the appropriate solvent. Data for ^{1}H NMR are reported as follows: chemical shift, multiplicity (br = broad, s = singlet, bs = broad singlet, d = doublet, t = triplet, m = multiplet), coupling constants, and integration. Tandem liquid chromatography/mass spectrometry (LCMS) was performed on a Waters 2795 separations module and 3100 mass detector, alternatively a Shimadzu LC-20AD separations module or Agilent 1200 series, with data acquired either directly on reaction mixtures or on purified samples. Tandem ultra-performance liquid chromatography/mass spectrometry (UPLC-MS) was performed on Waters Acquity UPLC systems with C18 columns (2.1×50 mm), PDA detectors, ELS detectors and Waters SQ mass detectors. Both instruments utilize a 3-minute 95:5→5:95 H_2O/CH_3CN gradient (0.9 mL/min flow rate) with either 0.05% CF_3COOH (acidic) or 0.1% NH_4OH (basic) additives.

Synthesis of BRD1389

((8*R*,9*R*,10*S*,*Z*)-9-(4-bromophenyl)-6-((4-nitrophenyl)sulfonyl)-1,6-diazabicyclo[6.2.0]dec-3-en-10-yl)methanol (2)

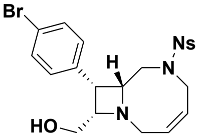

(8*R*,9*R*,10*S*,*Z*)-9-(4-bromophenyl)-6-((4-nitrophenyl)sulfonyl)-10-((trityloxy)methyl)-1,6-diazabicyclo[6.2.0]dec-3-ene (8.00 g, 10.7 mmol, 1.00 equiv) (1, prepared according to the method of Lowe, J. T. *et al.*)[1] was dissolved in CH_2Cl_2 (100 mL) and TFA was added (15.8 mL, 213 mmol, 20.0 equiv). The mixture was stirred at 15 °C for 6 h. After completion, the reaction was quenched by addition of sat. aq. $NaHCO_3$ until pH = 8, and then extracted with CH_2Cl_2 (3 × 30 mL). The combined organic layers were washed with brine (50 mL), dried over Na_2SO_4, filtered and concentrated *in vacuo*. The resulting mixture was partially purified by column chromatography (SiO_2, petroleum ether/ethyl acetate = 20:1 to 0:1) to afford a crude brown solid (3.80 g). A portion of this material was engaged in the next step without further purification.

(8*R*,9*R*,10*S*,*Z*)-9-(4-bromophenyl)-10-(methoxymethyl)-6-((4-nitrophenyl)sulfonyl)-1,6-diazabicyclo[6.2.0]dec-3-ene (3)

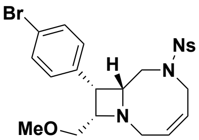

Alcohol 2 (crude, 1.00 g aliquot, 1.97 mmol, 1.00 equiv) was dissolved in DMF (10 mL). Sodium hydride (60% dispersion in mineral oil, 236 mg, 5.90 mmol, 3.00 equiv) was added dropwise at 0 °C under N_2. The reaction mixture was stirred at 0 °C for 1 h. Then iodomethane (1.12 g, 7.87 mmol, 489 µL, 4.00 equiv) was added and the mixture was heated to 25 °C and stirred for 11 h. After completion, the reaction was cooled to 0 ºC, quenched by addition of H_2O (10 mL), and extracted with EtOAc (3 × 30 mL). The combined

organic layers were dried over Na_2SO_4, filtered and concentrated *in vacuo*. The resulting mixture was partially purified by column chromatography (SiO_2, petroleum ether/ethyl acetate = 10:1 to 1:1) to afford a crude yellow oil (380 mg) engaged in the next step without further purification.

(8*R*,9*R*,10*S*,*Z*)-9-(4-bromophenyl)-10-(methoxymethyl)-1,6-diazabicyclo[6.2.0]dec-3-ene (4)

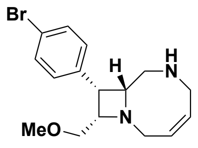

Sulfonamide 3 (crude, 380 mg, 0.727 mmol, 1.00 equiv) was dissolved in CH_3CN (10 mL). Cs_2CO_3 (474 mg, 1.45 mmol, 2.00 equiv) and benzenethiol (120 mg, 1.09 mmol, 111 µL, 1.50 equiv) were then added in one portion and the mixture was heated at 40 °C. After 2 h, the reaction was quenched by addition of H_2O (10 mL) and then extracted with CH_2Cl_2 (3 × 10 mL). The combined organic layers were dried over Na_2SO_4, filtered and concentrated *in vacuo*. The resulting mixture was partially purified by preparative TLC (SiO_2, petroleum ether/ethyl acetate = 0:1) to afford a crude yellow oil (160 mg). A portion of this material was engaged in the next step without further purification.

1-cyclopropoxy-4-isocyanatobenzene (5)

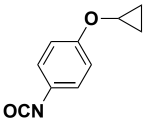

To a solution of bis(trichloromethyl) carbonate (19.7 mg, 67.0 µmol, 0.500 equiv) in toluene (0.260 mL) at 20 °C, under N_2, was added dropwise a solution of 4-(cyclopropoxy)aniline (19.8 mg, 0.133 mmol, 1.00 equiv, prepared according to the method of Aguilar, Nuria *et al.*)[2] in dioxane (47.0 µL). The resulting mixture was warmed to 110 °C and stirred for 1 h. During this period, the initial suspension turned into a clear mixture, which was concentrated *in vacuo* and used in the next step without further purification.

(8*R*,9*R*,10*S*,*Z*)-9-(4-bromophenyl)-*N*-(4-cyclopropoxyphenyl)-10-(methoxymethyl)-1,6-diazabicyclo[6.2.0]dec-3-ene-6-carboxamide (6)

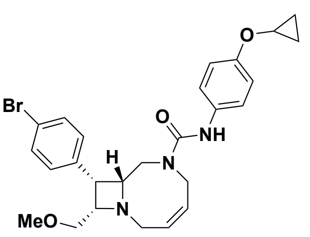

Amine 4 (crude, 30.0 mg aliquot, 89.0 μmol, 1.00 equiv) was dissolved in CH_2Cl_2 (1.77 mL). Et_3N (24.6 μL, 0.177 mmol, 2.00 equiv) and isocyanate 5 (23.2 mg, 0.133 mmol, 1.50 equiv, prepared as described above) were added at 0 °C under N_2. The mixture was stirred at 20 °C for 30 min and concentrated *in vacuo*. The residue was purified by column chromatography (SiO_2, ethyl acetate/hexane = 0:1 to 7:3) to afford the desired compound (24.2 mg, calculated yield: 8.9% from 1).

LC-MS m/z calculated for $C_{26}H_{30}BrN_3O_3Na$ $[M+Na]^+$ 534.15; Found 534.17.

^{1}H NMR (400 MHz, chloroform-*d*) δ 7.45 (d, *J* = 7.8 Hz, 2H), 7.35 (d, *J* = 8.1 Hz, 2H), 7.20 (d, *J* = 8.4 Hz, 2H), 6.94 (d, *J* = 8.4 Hz, 2H), 6.10 (s, 1H), 5.88 – 5.78 (m, 1H), 5.76 – 5.67 (m, 1H), 4.20 (d, *J* = 16.8 Hz, 1H), 3.98 (dd, *J* = 16.4, 7.3 Hz, 1H), 3.74 – 3.39 (m, 7H), 3.22 (d, *J* = 5.5 Hz, 1H), 3.16 (s, 4H), 2.85 (t, *J* = 12.4 Hz, 1H), 0.73 (d, *J* = 4.5 Hz, 4H).

(3*S*,4*R*,8*R*,9*R*,10*S*)-9-(4-bromophenyl)-*N*-(4-cyclopropoxyphenyl)-3,4-dihydroxy-10-(methoxymethyl)-1,6-diazabicyclo[6.2.0]decane-6-carboxamide (7a)

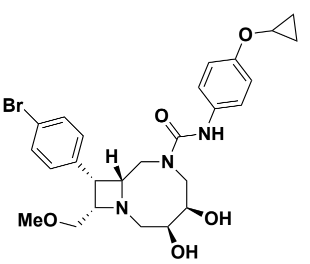

Olefin 6 (24.2 mg, 47.0 μmol, 1.00 equiv) was dissolved in acetone (0.393 mL) and water (76.0 μL). 4-methylmorpholine *N*-oxide (19.0 μL, 94.0 μmol, 2.00 equiv) and osmium(VIII) tetroxide solution (4 wt. % in H_2O, 2.90 μL, 0.470 μmol, 0.0100 equiv) were added at 25 °C, and the mixture was stirred at this temperature for 16 h. Next, the mixture was dried over Na_2SO_4, filtered and concentrated *in vacuo*. The resulting mixture was purified by flash column chromatography (SiO_2, ethyl acetate/hexane = 0:1 to 1:0) to afford the desired compound 7a (9.00 mg, yield: 35%) and its diastereomer 7b (see below, 13.1 mg, yield: 45%).

LC-MS m/z calculated for $C_{26}H_{32}BrN_3O_5Na$ $[M+Na]^+$ 568.15; Found 568.24.

^{1}H NMR (400 MHz, chloroform-d) δ 7.77 (bs, 1H), 7.43 (d, J = 8.0 Hz, 2H), 7.34 (d, J = 8.2 Hz, 2H), 7.18 (d, J = 8.4 Hz, 2H), 6.95 (d, J = 8.5 Hz, 2H), 4.29 (dd, J = 16.1, 5.5 Hz, 1H), 4.07 (d, J = 5.0 Hz, 1H), 3.91 – 3.59 (m, 5H), 3.56 – 3.37 (m, 3H), 3.29 – 3.17 (m, 2H), 3.15 (s, 3H), 2.95 – 2.84 (m, 1H), 2.83 – 2.74 (m, 1H), 2.68 (t, J = 12.5 Hz, 1H), 2.29 (bs, 1H), 0.73 (d, J = 4.4 Hz, 4H).

(3*R*,4*S*,8*R*,9*R*,10*S*)-9-(4-bromophenyl)-*N*-(4-cyclopropoxyphenyl)-3,4-dihydroxy-10-(methoxymethyl)-1,6-diazabicyclo[6.2.0]decane-6-carboxamide (7b)

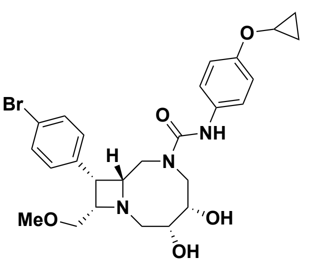

LC-MS m/z calculated for $C_{26}H_{32}BrN_3O_5Na$ $[M+Na]^+$ 568.15; Found 568.24.

^{1}H NMR (400 MHz, chloroform-*d*) δ 7.49 (d, *J* = 8.0 Hz, 2H), 7.35 – 7.24 (m, 4H), 6.98 (d, *J* = 8.5 Hz, 2H), 6.60 (bs, 1H), 3.87 (d, *J* = 7.0 Hz, 1H), 3.81 – 3.58 (m, 6H), 3.57 – 3.39 (m, 3H), 3.35 – 3.23 (m, 2H), 3.20 (s, 3H), 2.88 (bs, 1H), 2.82 (d, *J* = 14.1 Hz, 1H), 1.46 (d, *J* = 23.2 Hz, 1H), 0.76 (d, *J* = 4.5 Hz, 4H), 1 exchangeable proton not observed.

(3*S*,4*R*,8*R*,9*R*,10*S*)-*N*-(4-cyclopropoxyphenyl)-3,4-dihydroxy-10-(methoxymethyl)-9-(4-(phenylethynyl)phenyl)-1,6-diazabicyclo[6.2.0]decane-6-carboxamide (BRD1389)

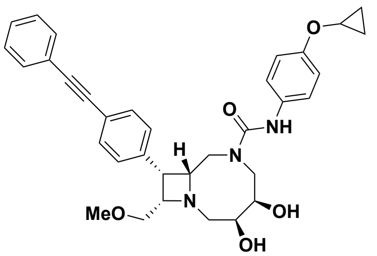

A sealed vial containing aryl bromide 7a (22.1 mg, 40.0 µmol, 1.00 equiv) was evacuated and backfilled with N_2 (× 3) then were added CH_3CN (0.400 mL, previously sparged with argon for 40 min), NEt_3 (22.3 µL, 0.161 mmol, 4.00 equiv) and phenylacetylene (22.1 µL, 0.202 mmol, 5.00 equiv), followed by XPhos-Pd-G3 (3.40 mg, 4.00 µmol, 0.100 equiv).

The vial was sealed and heated to 70 ºC. After 90 min, the reaction was allowed to cool at room temperature, sat. aq. $NaHCO_3$ was added, and the mixture was extracted with CH_2Cl_2 (3 × 0.40 mL). The combined organic layers were dried over Na_2SO_4, filtered and concentrated *in vacuo*. The residue was purified by flash column chromatography reverse phase (C18, CH_3CN/water = 0:1 to 1:1) to afford BRD1389 (16.0 mg, yield: 70%).

LC-MS m/z calculated for $C_{34}H_{38}N_3O_5$ $[M+H]^+$ 568.26; Found 568.86.

^1H NMR (400 MHz, chloroform-*d*) δ 7.73 (bs, 1H), 7.58 – 7.42 (m, 6H), 7.40 – 7.31 (m, 3H), 7.19 (d, *J* = 8.5 Hz, 2H), 6.95 (d, *J* = 8.8 Hz, 2H), 4.30 (dd, *J* = 16.0, 5.5 Hz, 1H), 4.12 – 4.05 (m, 1H), 3.94 – 3.74 (m, 3H), 3.72 – 3.61 (m, 2H), 3.56 (t, *J* = 7.6 Hz, 1H), 3.48 (dd, *J* = 10.1, 5.6 Hz, 2H), 3.32 – 3.20 (m, 2H), 3.15 (s, 3H), 2.92 (dd, *J* = 13.6, 9.2 Hz, 1H), 2.77 (dd, *J* = 26.0, 13.1 Hz, 2H), 2.33 (s, 1H), 0.73 (d, *J* = 4.4 Hz, 4H).

Supplementary Figures

Supplementary Figure 1

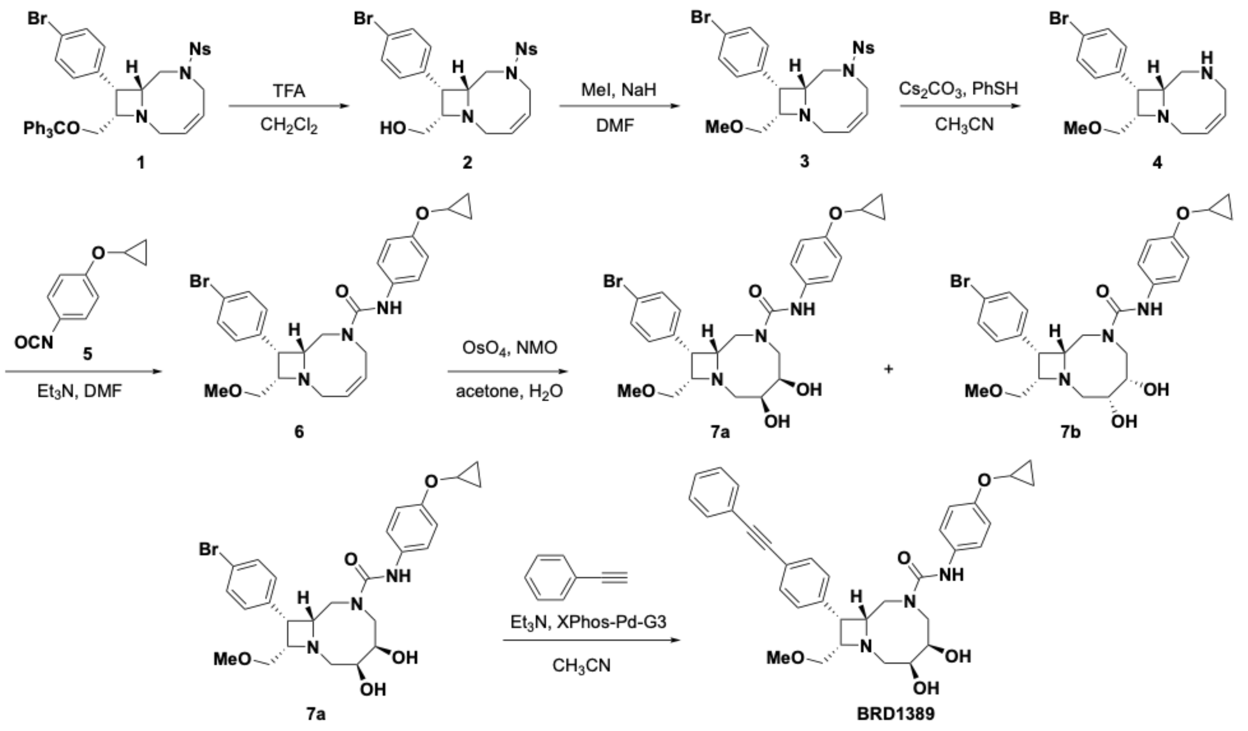

Supplementary Figure 1. Chemical synthesis of BRD1389.

Supplementary Figure 2

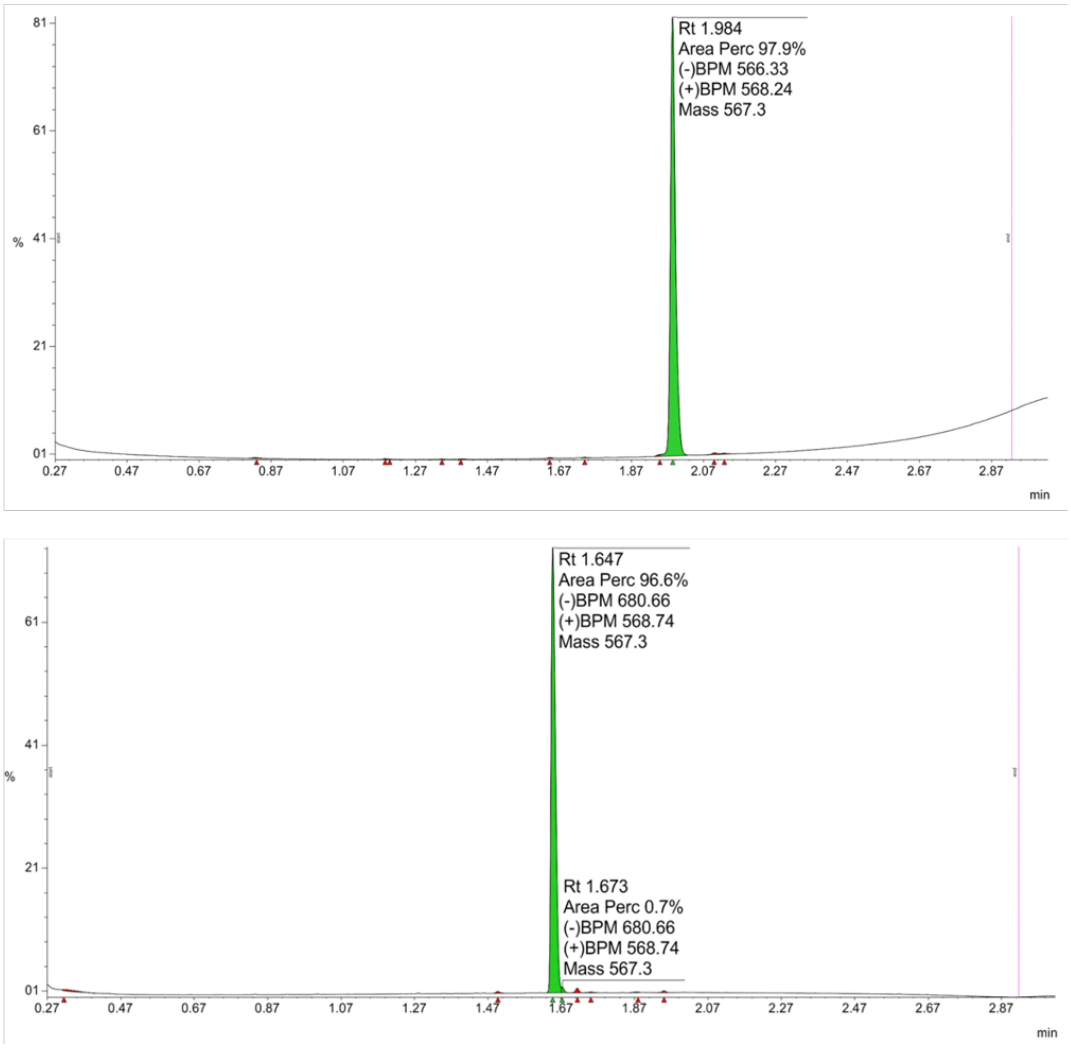

Supplementary Figure 2. Assessment of purity of BRD1389 by tandem ultraperformance liquid chromatography/mass spectrometry (UPLC-MS) using mobile phases containing basic **(top)** and acid **(bottom)** additives. The peaks matching the exact mass of BRD1389 are highlighted in green, and peak retention times, relative integrations, and observed masses in positive and negative ionization modes are indicated.

Supplementary Figure 3

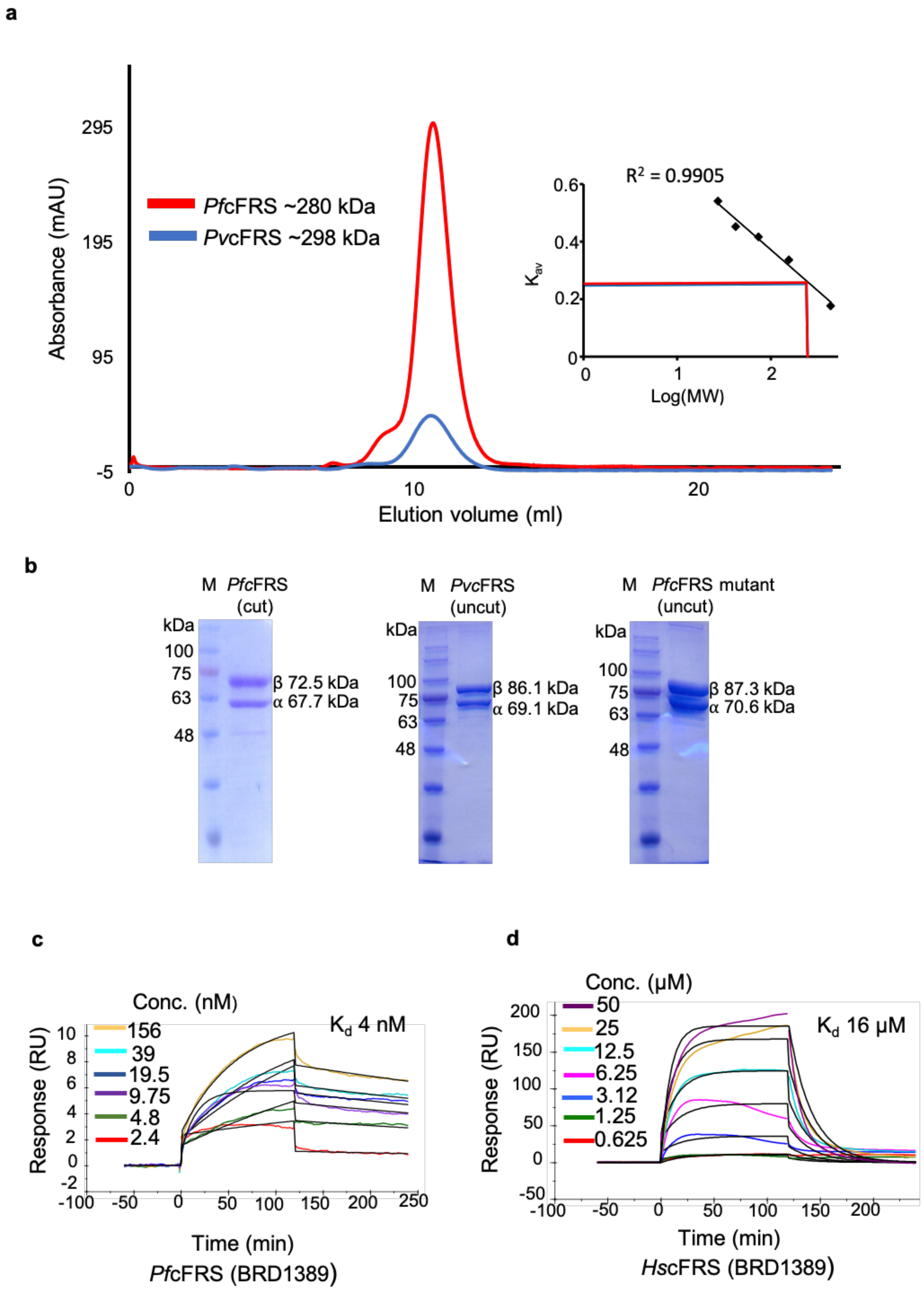

Supplementary Figure 3. (a) GPC elution profiles of *Pf*cFRS (blue) and *Pv*cFRS (red). Comparison with standard markers suggests that the *Pf*cFRS and *Pv*cFRS elute at a size that corresponds to that of a tetramer. **(b)** SDS-PAGE profile of purified proteins *Pfc*FRS (cut; α-67.7 kDa, β-72.5 kDa), *Pvc*FRS (uncut; α-69.1 kDa, β-86.1 kDa) and *Pfc*FRS mutant L550V (uncut; α-70.6 kDa, β-87.3 kDa). Source data are provided as a Source Data file. **(c, d)** Sensogram showing the binding of BRD1389 with *Pf*cFRS and *Hs*cFRS enzymes.

Supplementary Figure 4

a b

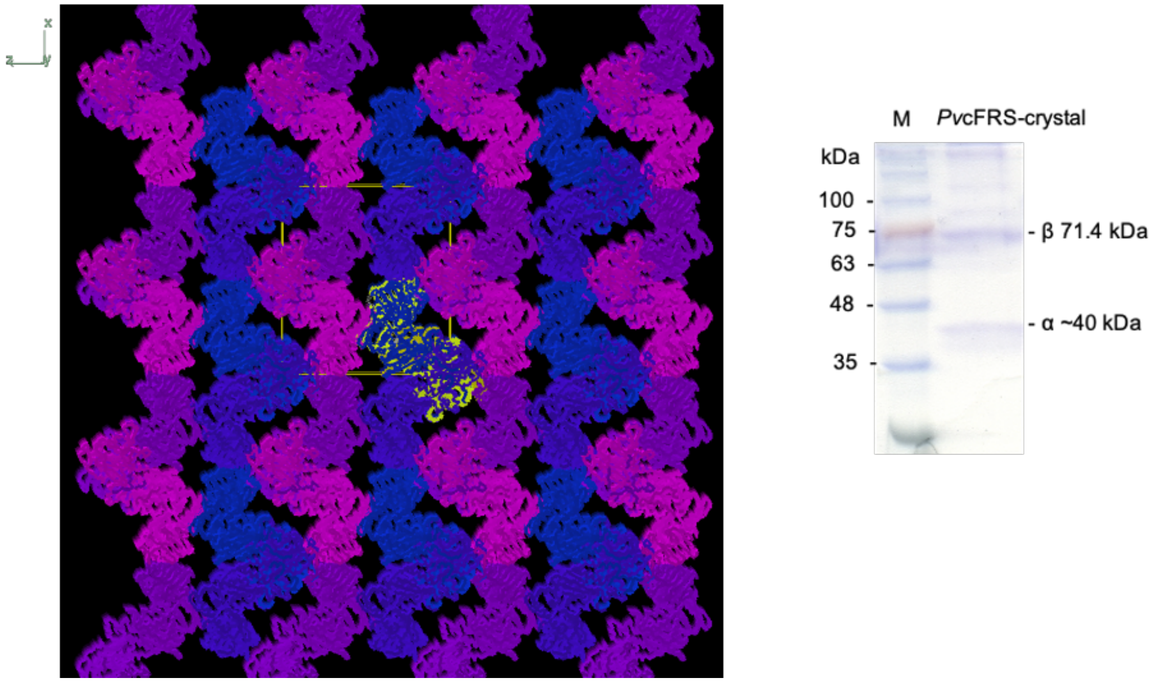

Supplementary Figure 4. a) A view along the b-axis of *Pvc*FRS-BRD1389 complex crystal showing lack of space for the N-terminal stretch (1-270) of the alpha subunit. **b)** SDS-PAGE analysis of the crystallized *Pvc*FRS protein showing the reduced size of α-subunit (40 kDa). Source data are provided as a Source Data file.

Supplementary Figure 5

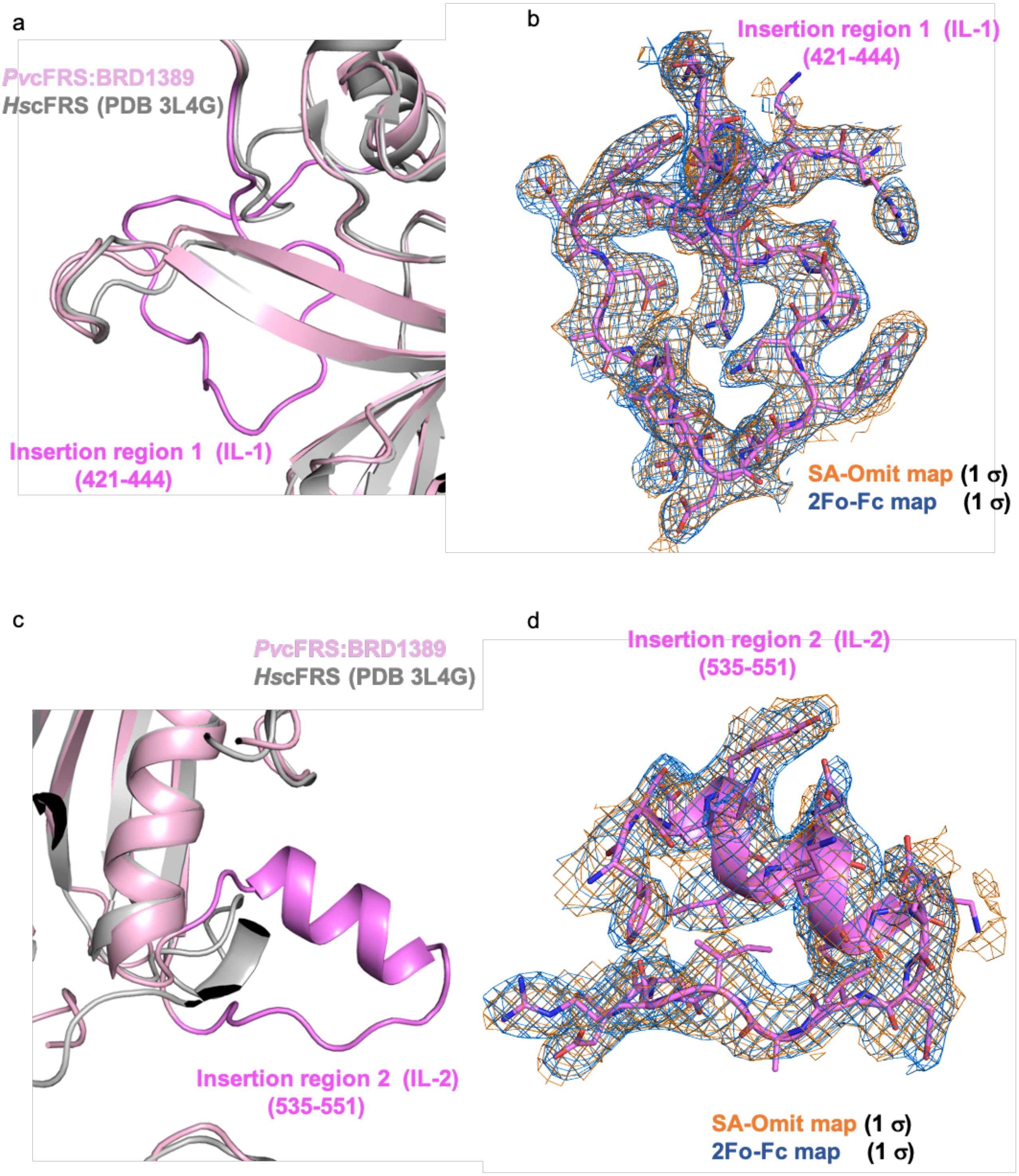

Supplementary Figure 5. a) A closeup view of the insertion region 1 (IL1) (residues 421-444, dark pink) in β2 sub-domain of beta subunit in *Pv*FRS-BRD1389 complex (pink) and its comparison with *Hs*FRS (grey). **b)** The composite simulated annealed omit (SA-omit, orange) and final 2Fo-Fc (blue) maps are contoured at 1 σ levels (right) for insertion region IL1. **c)** Close up view of the unique insertion 2 (IL2) region (residues 535-551, dark pink) in B2 sub-domain of beta subunit within the *Pv*FRS-BRD1389 complex (pink) and its comparison with *Hs*FRS (grey) (right panel). **d)** The composite simulated annealed omit (SA-omit, orange) and final 2Fo-Fc (blue) maps are contoured at 1 σ levels (right) for the insertion region IL2.

Supplementary Figure 6

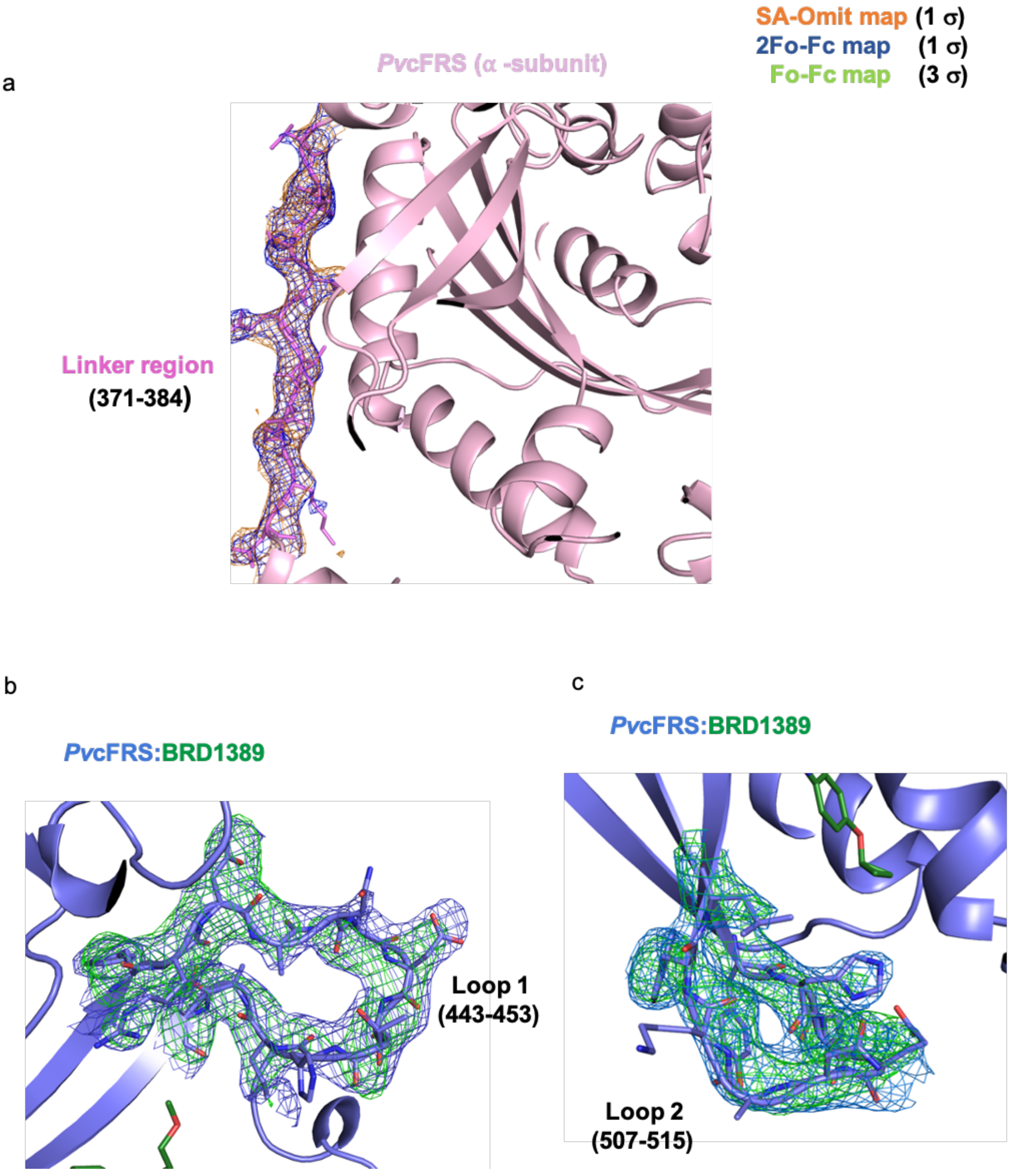

Supplementary Figure 6. **a)** Closeup view of the linker region between β1 and β2 sub-domains of beta subunit in *Pv*FRS-BRD1389 complex with a composite simulated annealed omit (SA-omit, orange) and final 2Fo-Fc (blue) maps contoured at 1 σ levels. **b)** The difference final (2Fo-Fc, blue) and difference Fourier (Fo-Fc, green) maps are contoured at 1 σ and 3 σ levels respectively for the active site loop 1. **c)** The difference final (2Fo-Fc, blue) and difference Fourier (Fo-Fc, green) maps are contoured at 1 σ and 3 σ levels respectively for active site loop 2.

Supplementary Figure 7

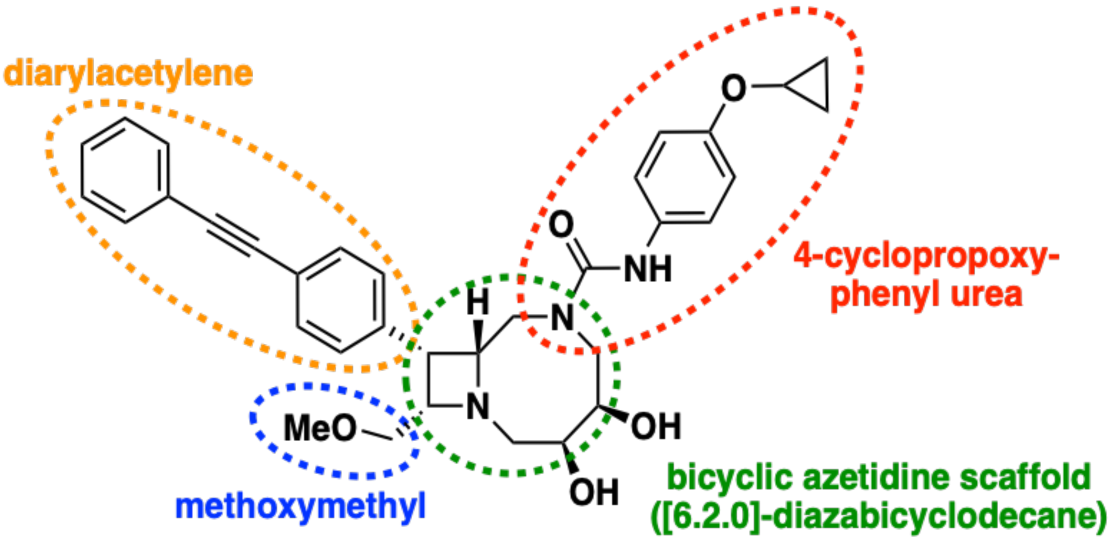

BRD1389

Supplementary Figure 7. Rationalised chemical structure of BRD1389 highlighting its [6.2.0]-diazabicyclodecane core (circled in green), its 4-cyclopropoxyphenyl (red), diarylacetylene (orange) and methoxymethyl (blue) appendages.

Supplementary Figure 8

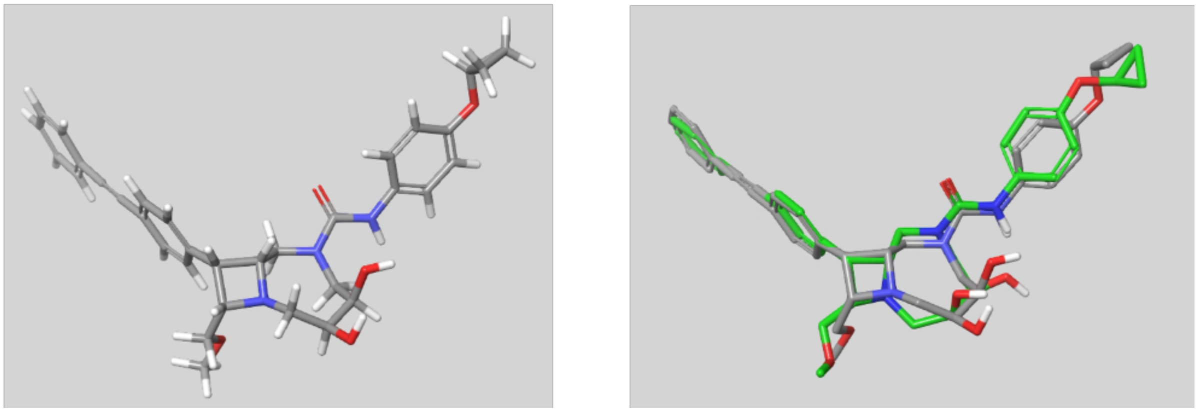

Supplementary Figure 8. Left: Computed optimal conformation of BRD1389 in water. Right: Superimposed computed solution structure (grey) and crystallographic *Pv*cFRS-bound structure (green). Polar hydrogen atoms are omitted for clarity and the root mean square deviation (rmsd) is 1.1911 Å.

Supplementary Figure 9

```
                                          PfM316I
Pf        KNEEYKKYDIKKYNFFSSGKKINKGNIHLLTKQMRTFKEIFFSLGFEEMETHNYVESSFW     342
Pv        KNEEYKKYQVKKYNFFSSGKKMNKGNIHLLIRQMRTFKDVFVSLGFEEMNTHNYVESSFW     336
Po        RNDEYAKYDIKKYNFFSSGKKMNKGNIHLLTRQMRAFKDIFISLGFEEMDTQNYVESSFW     319
Pm        KNEEYKKYDIKEYNFFSSGKKIIKGTLHVLTKQMRIFKDIFISLGFEEMETHNYVESSFW     336
Hs        SSGSWRDRPFKPYNFLAHGVLPDSGHLHPLLKVRSQFRQIFLEMGFTEMPTDNFIESSFW     257
Crypto    MNSSWENNEFKPYNFNAKGKRLPRGNIHPLTRTSRKFKRILSQMGFEEMPTNRWVESSFW     267
Tg        IGDAWEKSNFKEYNFFAAGKRIRRGAVHPLMQVMKQFKQILYCMGFEEMPTNQYVESSFW     289

Pf        CFDALYIPQQHPSRDLQDTFFIKEPETCIDKF-VDTEYIDNIKRVHTHGDYGSFGWNYKW     401
Pv        CFDALYIPQQHPSRDLQDTFFIKVPEMCQEEF-TDQSYIENVKRVHSVGDYGSFGWNYQW     395
Po        CFDALYIPQQHPSRDLQDTFFIKEPEMCADNF-IDSDYIANIEKVHTYGDYGSFGWNYKW     378
Pm        CFDALYIPQQHPSRDLQDTFFIKDPEICQDNF-IDIGYIENVKRVHSVGDYGSFGWNYEW     395
Hs        NFDALFQPQQHPARDQHDTFFLRDPAEA---LQLPMDYVQRVKRTHSQGGYGSQGYKYNW     314
Crypto    NFDALFQPQKHPARDSHDTFFLETPSTFRNEMELSNDHIDKVKKVHEVGGYGSIGLSYNW     327
Tg        CFDSLFMPQQHPARDVQDTFFLQAPKSSDATK-IPQAYFNSVKNIHERGGHGSIGWQYEW     348

Pf        KLEESKKNVLRTHTTANSCRALFKLAKEYKEA--GC----IKPKKYFSIDRVFRNENLDS     455
Pv        ELKSTKKNVLRTHTTANSCRALFQLAKEYQKT--GS----IIPKKFFSIDRVFRNENLDS     449
Po        KLEESKKNVLRTHTTANSCRTLFKLAKEYNKK--GC----IIPKKYFSIDRVFRNENLDS     432
Pm        KIEESKRNVLRTHTTSNSCRTLFKLAKEYNRK--GH---IILPKKYFSIDRVFRNENLDS     450
Hs        KLDEARKNLLRTHTTSASARALYRLAQKKP----------FTPVKYFSIDRVFRNETLDA     364
Crypto    SIEEASKNLLRTHTTAVSVRMLYKLAQMYLYNENGLSFDNFQRKAYFSIDRVFRNESIDA     387
Tg        SEEESMGNILRTHTTASSARMLYGLAQEYKKT--GV----FRPRKFFSIDRVFRNETLDA     402

                                                             PfG512E
Pf        THLAEFHQVEGLIIDKNIGLSHLIGTLAAFYKHIGIHKLKFKPTFNPYTEPSMEIYGYHE     515
Pv        THLAEFHQVEGLIIDRNLGLSHLIGTLSAFYKYIGISKLRFKPTFNPYTEPSMEVYGYHE     509
Po        THLAEFHQIEGLIIDKNLGLSQLISTLSAFYKYIGIHKLKFKPTFNPYTEPSMEIYGYHE     492
Pm        THLAEFHQVEGLIIDKNLGLAHLIGTLSAFYKYIGIHKLKFKPAFNPYTEPSMEIFGYHE     510
Hs        THLAEFHQIEGVVADHGLTLGHLMGVLREFFTKLGITQLRFKPAYNPYTEPSMEVFSYHQ     424
Crypto    THLAEFHQVEGLIVDKNLTLADLIGTLKTFYEKIGISDLKFKPAFNPYTEPSMEIFGYHT     447
Tg        THLAEFHQVEGLVADRGLTIGHLMGVMETFYKQIGIEQLKFKPAYNPYTEPSMEIFGYHA     462

                                         PfV545I PfL550V
Pf        QSKKWLEVGNSGIFRPEMLRSMGFSEEVSVIAWGLSLERPTMIKYNIKNIRDLFGYKSVV     575
Pv        ENKKWLEVGNSGIFRPEMLRAMGFPPEVSVIAWGLSLERPTMIKYSIRNIRDLFGYRSVI     569
Po        ENKKWLEVGNSGVFRPEMLRSMGFEKDVSVIAWGLSLERPTMIKYNIKNIRDLFGYKSVV     552
Pm        QSRKWLEVGNSGVFRPEMLRAMGFPKDVSVIAWGLSLERPTMIKYNVKNIRDLFGYRSTL     570
Hs        GLKKWVEVGNSGVFRPEMLLPMGLPENVSVIAWGLSLERPTMIKYGINNIRELVGHKVNL     484
Crypto    TLKKWIEVGNSGLFRPEMLRPLGFPSDIVVIAWGLSLERPTMISYNIPNIRDLFSFKAKI     507
Tg        GLKRWIEVGNSGIFRPEMLLPMGLPEDVTVIAWGLSLERPTMIRYGISNIRQLFGHRALL     522
```

Supplementary Figure 9. Sequence alignment of α subunit of FRSs from various eukaryotic pathogens and their human counterpart. Residues forming the L-Phe pocket are in bold; residues that vary within a 5 Å radius of the drug amongst species are shown in red; black boxes indicate resistance mutations identified in *Pf*cFRS under selection pressure using bicyclic azetidine BRD1095[2]; orange boxes indicate non-conserved residues within 5 Å of the site that may be responsible for selectivity. *Pf, Plasmodium falciparum; Pv, Plasmodium vivax; Po, Plasmodium ovale; Pm, Plasmodium malariae, Hs, Homo sapiens; Crypto; Cryptosporidium parvum, Tg; Toxoplasma gondii.*

Supplementary Tables

Supplementary Table 1. Data collection and refinement statistics.

	PvFRS:BRD1389 binary complex
PDB code	7BY6
**Data collection**	
Beamline	I24
Wavelength (Å)	0.9688
Detector type	PILATUS3 6M, S/N 60-0119
Crystal-to-detector distance (mm)	580
Oscillation (°)	0.1
Exposure (s)	0.020
Beam size (μm)	50 x 50
Flux (photons s^{-1})	2.30 e^{+0}
Transmission (%)	100
No. of images	1800
Software used for data processing	*xia2*
Space group	P 2_1 2_1 2
Cell dimensions<br>a, b, c (Å)<br>α, β, γ (°)	 136.52, 74.07, 121.68<br>90.0, 90.0, 90.0
Resolution (Å)	121.78-3.00 (3.05-3.00)*
R_{meas} (%)	0.145 (1.253)
I/σI	8.4 (1.7)
Completeness (%)	99.8 (95.0)
Redundancy	6.3 (6.3)
$CC_{1/2}$	0.9 (0.8)
No. of unique reflections	25457 (1191)
**Refinement**	
Resolution (Å)	121.78-3.00 (3.12-3.0)
No. of reflections /test set	25375 /1296
R_{work}/ R_{free} (%)	21.4/28.8
No. of protein residues	Chain A: 299 /Chain B: 606
No. atoms	
Protein	Chain A: 2365 /Chain B: 4516
Ligand FB9 (non-H atoms) / Mg^{2+} ion	42 /1
Average B-factors ($Å^2$)	
Protein	93.3
Ligand FB9 / Mg^{2+} ion	68.8 / 83.1
R.m.s deviations	
Bond lengths (Å)	0.009
Bond angles (º)	1.135

Ramachandran plot	
Favoured/Allowed (%)	92.0/8.0

Supplementary Table 2. Optimized geometry and computed energy of BRD1389 in water.

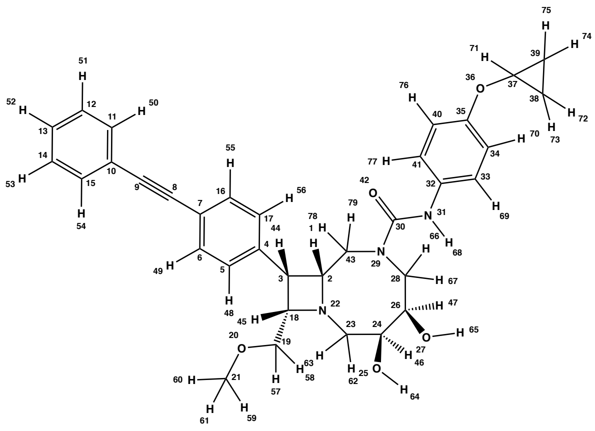

Atom	Coordinate (Å)		
	X	Y	Z
H1	1.2953	3.3898	2.1982
C2	0.2493	3.2079	2.4897
C3	-0.5852	4.5364	2.4896
C4	-1.6999	4.7921	1.4983
C5	-1.4906	5.8058	0.5442
C6	-2.4116	6.0718	-0.4614
C7	-3.602	5.3236	-0.5529
C8	-4.5235	5.5815	-1.6123
C9	-5.267	5.8243	-2.5446
C10	-6.1097	6.1215	-3.6589
C11	-7.3337	5.4467	-3.8459
C12	-8.1374	5.7392	-4.9462
C13	-7.7389	6.7036	-5.8761
C14	-6.5288	7.3806	-5.6979
C15	-5.7185	7.0961	-4.6012
C16	-3.8337	4.3227	0.4122
C17	-2.9006	4.0636	1.4148
C18	-0.7325	4.3255	4.0433
C19	-2.0842	4.1911	4.7389
O20	-2.782	3.0199	4.3425
C21	-4.0872	2.9597	4.9011

N22	0.0691	3.0842	3.9522
C23	1.2343	2.8853	4.7977
C24	1.4286	1.4283	5.261
O25	2.5187	1.3886	6.1993
C26	1.6954	0.3462	4.1934
O27	2.9557	0.6577	3.5812
C28	0.5429	0.0876	3.1661
N29	0.6024	0.82	1.8991
C30	1.4646	0.5041	0.8712
N31	2.0702	-0.7388	0.9566
C32	3.0404	-1.2577	0.071
C33	3.1665	-2.6552	-0.0175
C34	4.1411	-3.2358	-0.8213
C35	5.0092	-2.4298	-1.5666
O36	5.9394	-3.0942	-2.3393
C37	6.7986	-2.3134	-3.148
C38	8.1225	-2.9386	-3.4558
C39	8.044	-1.734	-2.5342
C40	4.8899	-1.0391	-1.4905
C41	3.9163	-0.4578	-0.6745
O42	1.6626	1.2754	-0.0832
C43	-0.2588	1.9973	1.7172
H44	0.1238	5.3572	2.3539
H45	-0.2137	5.14	4.5729
H46	0.5315	1.1268	5.8146
H47	1.7963	-0.5839	4.7689
H48	-0.5769	6.3944	0.587
H49	-2.2152	6.8549	-1.1877
H50	-7.6443	4.6959	-3.1258
H51	-9.0776	5.2109	-5.08
H52	-8.3676	6.9269	-6.734
H53	-6.2146	8.1317	-6.4173
H54	-4.7776	7.621	-4.4634
H55	-4.7507	3.7417	0.3655
H56	-3.1074	3.2967	2.1514
H57	-2.6802	5.0941	4.5204
H58	-1.92	4.1725	5.8301
H59	-4.5415	2.0223	4.5691
H60	-4.057	2.972	6.0015

H61	-4.7147	3.7993	4.5636
H62	1.1172	3.4813	5.7103
H63	2.1599	3.2347	4.3072
H64	3.3302	1.4018	5.6593
H65	3.4337	-0.1708	3.4148
H66	0.4934	-0.99	2.9709
H67	-0.4066	0.3437	3.6437
H68	1.6461	-1.4272	1.5654
H69	2.4923	-3.292	0.5515
H70	4.2344	-4.3166	-0.8844
H71	6.2807	-1.7529	-3.9257
H72	8.5238	-2.8139	-4.4577
H73	8.3308	-3.9029	-2.9984
H74	8.1996	-1.9079	-1.4728
H75	8.3905	-0.7685	-2.8924
H76	5.56	-0.3978	-2.0514
H77	3.8373	0.62	-0.6217
H78	-1.2667	1.739	2.0575
H79	-0.2945	2.2218	0.6501
**Energy** (solution phase)		–1858.22081138481 Hartree	

Supplementary Table 3. Table shows equivalent *Pf*cFRS resistance residues under drug pressure (in red) and wild type residues (in black) . The amino acid residues (in black) at the corresponding positions in *Pvc*FRS and the *Hs*cFRS are also listed.

**PvcFRS**	**P*f*cFRS**	***Hs*cFRS**
M (310)	M - I (316)	R (231)
G (506)	G - E (512)	S (421)
V (539)	V - I (545)	V (454)
L (544)	L - V (550)	L (459)

References

1. Lowe, J. T. *et al.* Synthesis and profiling of a diverse collection of azetidine-based scaffolds for the development of CNS-focused lead-like libraries. *J. Org. Chem.* 2012, *77*, 7187–7211.
2. Aguilar, N.; Fernandez, J. C.; Terricabras, E.; Carceller Gonzalez, E.; Garcia Garcia, F. J.; Salas Solana, J. Substituted Tricyclic Compounds with Activity Towards EP1 Receptors. WO2013149996.
